# Draft Genome Sequence of *Curtobacterium* sp. Strain ER1/6, an Endophytic Strain Isolated from *Citrus sinensis* with Potential To Be Used as a Biocontrol Agent

**DOI:** 10.1128/genomeA.01264-16

**Published:** 2016-11-17

**Authors:** Leandro Maza Garrido, João Marcelo Pereira Alves, Liliane Santana Oliveira, Arthur Gruber, Gabriel Padilla, Welington Luiz Araújo

**Affiliations:** a NAP/BIOP, Departamento de Microbiologia, Instituto de Ciências Biomédicas, Universidade de São Paulo, Biomédicas II, Cidade Universitária, São Paulo, São Paulo, Brazil; b Departamento de Parasitologia, Instituto de Ciências Biomédicas, Universidade de São Paulo, Biomédicas II, Cidade Universitária, São Paulo, São Paulo, Brazil

## Abstract

Herein, we report a draft genome sequence of the endophytic *Curtobacterium* sp. strain ER1/6, isolated from a surface-sterilized *Citrus sinensis* branch, and it presented the capability to control phytopathogens. Functional annotation of the ~3.4-Mb genome revealed 3,100 protein-coding genes, with many products related to known ecological and biotechnological aspects of this bacterium.

## GENOME ANNOUNCEMENT

The plant environment is an important habitat for *Curtobacterium* species that may interact with their host plant, inducing disease symptoms or controlling phytopathogens. This genus was first described by Yamada and Komagata (1) and includes eight recognized *Curtobacterium* species (*C. albidum, C. ammoniigenes, C. citreum*, *C. flaccumfaciens, C. ginsengisoli, C. herbarum*, *C. luteum*, and *C. pusillum*) within the phylum *Actinobacteria*. The citrus endophytic *Curtobacterium* sp. strain ER1/6 was isolated from *Citrus sinensis* (sweet orange) and previously identified by fatty acid methyl ester (FAME-MIDI) as *C. flaccumfaciens* (2). Further analysis showed that this strain is a divergent genotype which lacks the ability to induce disease symptoms in beans and ornamental seedlings. In addition, a previous study showed that this endophytic bacterium may inhibit the citrus variegated chlorosis (CVC) symptoms caused by *Xylella fastidiosa* (3). Thus, based on its phenotypic and physiological properties, as well as phylogenetic distinctiveness, this citrus endophytic strain might represent a novel species in the *Curtobacterium* genus.

The genomic DNA, isolated from an overnight culture in tryptic soy broth (TSB) using a commercial Wizard genomic DNA purification kit (Promega Co.), was sequenced using the Illumina HiSeq 2000 platform in order to reach about 300-fold depth of coverage. A total of 1,074,582,228 bp were generated from a 100-bp paired-end run, with 97.5% of bases showing quality scores above 20. Paired-end data sets merging, trimming, and *de novo* assembly were performed using Geneious (version 7.1.9; Biomatters). The final assembly comprised 19 large contigs (>1,000 bp) totaling 3,368,973 bp in length and presenting a G+C content of 72.2%.

The assembled genomic sequence was submitted to a functional annotation pipeline constructed with the EGene2 platform (4). Gene predictions were performed using Glimmer 3.02 (5), tRNAscan-SE 1.3 (6), and RNAmmer 1.2 (7). Translated products were submitted to several analyses, including BLAST similarity searches against NCBI’s nonredundant (nr) database, identification of protein motifs on InterPro (8), and Conserved Domain Database (CDD) (9), orthology mapping using eggNOG (10) and KEGG Orthology (KO), KEGG pathway mapping (11), and GO (Gene Ontology) term mapping (12). We found a total 3,239 genes, including 3,100 protein-coding genes, 87 noncoding RNAs (ncRNAs), and 47 tRNAs, comprising a complete set for the 20 amino acids. In addition, genes for rRNAs of the 5S, 16S and 23S subunits were also found. A total of 1,259 and 2,279 protein sequences were successfully classified in KO and eggNOG orthologous groups, respectively. A survey of secondary metabolites pathways using antiSMASH 2.0 (13) revealed six putative gene clusters (two terpenes, one type III-polyketide synthases [PKS], one siderophore, one bacteriocin, and one unknown).

### Accession number(s).

This communication describes the first version of a whole-genome shotgun project of the citrus endophytic *Curtobacterium* strain ER1/6. The nucleotide sequence and annotation data have been deposited at DDBJ/EMBL/GenBank under the accession number MJAK00000000.
